# Does co-presence affect the way we perceive and respond to emotional interactions?

**DOI:** 10.1007/s00221-020-06020-5

**Published:** 2021-01-11

**Authors:** Julia Bachmann, Adam Zabicki, Stefan Gradl, Johannes Kurz, Jörn Munzert, Nikolaus F. Troje, Britta Krueger

**Affiliations:** 1 NeuroMotor Behavior Lab, Department of Psychology and Sport Science, Justus-Liebig-University, Giessen, Germany; 2 Machine Learning and Data Analysis Lab, Faculty of Engineering, Friedrich-Alexander-University Erlangen-Nuremberg, Erlangen, Germany; 3 Center for Mind, Brain and Behavior (CMBB), Philipps University of Marburg and Justus Liebig University, Giessen, Germany; 4 BioMotionLab, Department of Biology and Centre for Vision Research, York University Toronto, Toronto, Canada

**Keywords:** Emotion perception, Co-presence, Implicit response behavior, Explicit response behavior, Gender differences

## Abstract

This study compared how two virtual display conditions of human body expressions influenced explicit and implicit dimensions of emotion perception and response behavior in women and men. Two avatars displayed emotional interactions (angry, sad, affectionate, happy) in a “pictorial” condition depicting the emotional interactive partners on a screen within a virtual environment and a “visual” condition allowing participants to share space with the avatars, thereby enhancing co-presence and agency. Subsequently to stimulus presentation, explicit valence perception and response tendency (i.e. the explicit tendency to avoid or approach the situation) were assessed on rating scales. Implicit responses, i.e. postural and autonomic responses towards the observed interactions were measured by means of postural displacement and changes in skin conductance. Results showed that self-reported presence differed between pictorial and visual conditions, however, it was not correlated with skin conductance responses. Valence perception was only marginally influenced by the virtual condition and not at all by explicit response behavior. There were gender-mediated effects on postural response tendencies as well as gender differences in explicit response behavior but not in valence perception. Exploratory analyses revealed a link between valence perception and preferred behavioral response in women but not in men. We conclude that the display condition seems to influence automatic motivational tendencies but not higher level cognitive evaluations. Moreover, intragroup differences in explicit and implicit response behavior highlight the importance of individual factors beyond gender.

**Supplementary Information:**

The online version contains supplementary material available at 10.1007/s00221-020-06020-5.

## Introduction

Recognizing human body expressions is vital for responding appropriately to social cues. Bodily expressions do not just convey a person’s affective states, they also inform about action demands. For instance, a fearful face tells how someone is feeling, but it does not necessarily indicate how to respond. However, if a person reacts fearfully with their whole body by, for instance, drawing away from a potential threat, one can prepare for a concrete action. Evolutionary psychology considers emotions to be action dispositions that humans possess to navigate in the world (Bradley et al. 2001; de Gelder 2006; de Gelder et al. 2015; LeDoux 1996). Different tools have been used to study the perception of emotional body language. Some of the most common include video displays, stick figures, and point-light displays—that is, displays depicting biological motion by the kinematics of light points located on an actor’s joints (see, e.g., Atkinson et al. 2004; Kaletsch et al. 2014a, b; Krüger et al. 2018; Lorey et al. 2012). There is evidence that humans are able to quickly identify a person’s affective state even from highly impoverished stimulus material, thus demonstrating the importance of top-down knowledge in enabling people to quickly make sense of what they are seeing.

This process of recognizing emotions is thought to be followed by a motivation to respond to them that generates so-called avoid-approach behavior (Bradley et al. 2001; Stins et al. 2011). This is based on the idea that positively valenced stimuli elicit approach tendencies, whereas negative stimuli elicit avoidance tendencies (e.g., Chen and Bargh 1999; Seidel et al. 2010b). These basal motivational tendencies can be separated into implicit and explicit stages. From an evolutionary perspective, implicit response tendencies are characterized by an automatic evaluation of incoming stimuli without conscious effort to quickly generate behavioral responses (Elliot and Covington 2001). Explicit response tendencies, in contrast, may represent more complex cognitive evaluations of a stimulus that occur at a later stage, and may potentially be affected more by social learning and reinforcement. Implicit response tendencies can, for instance, be operationalized via postural displacement. For example, Stins and Beek (2007) observed an increase in anterior center of pressure (COP) displacement in response to unpleasant images. Other researchers, in contrast, found a decrease in body sway during the presentation of unpleasant pictures. They interpreted this as freezing behavior, highlighting that the defensive system presents two basal dispositions: freezing or action (Azevedo et al. 2005; Facchinetti et al. 2006).

Many of these paradigms, however, are based on static or dynamic picture and video displays. Such “pictorial designs” separate observers physically through a screen without them being able to experience another’s presence—something that may be crucial for social cognition. Virtual environments offer promising tools with which to create vivid and realistic perceptual experiences by allowing for a sense of presence within virtual space (Seidel et al. 2010b; Slater 2009). Creating a sense of presence depends on valid sensorimotor contingencies between own body movements and the resulting changes in visual input. These sensorimotor contingencies are then thought to create the embodiment in space or “place illusion” (Slater 2009). Then, one is in “presence mode” compared to “picture mode” (Troje 2019). If the space in which one is embodied is also shared by other (virtual) agents, it is called “co-presence.” When in presence mode, the visual system seems to be in a similar state to when it is when interacting with the real world. This leads to an activation of the perceptual, vestibular, proprioceptive, and autonomic nervous systems in ways similar to those in a real-life situation (Slater 2003, 2009; Troje 2019).

Whereas the experimental setting may influence how we perceive emotions and how we respond to them, a considerable amount of research has demonstrated that emotion perception and response behavior can also be modulated by characteristics of the individual such as gender (Alaerts et al. 2011; Bradley et al. 2001; Hillman et al. 2004; Hoffmann et al. 2010). Although evidence is highly heterogeneous, studies suggest that women recognize emotions better than men (Alaerts et al. 2011; Hoffmann et al. 2010; Thayer and Johnsen 2000). More specifically, it has been shown that women detect emotions both more quickly and more accurately from facial or bodily expressions (Alaerts et al. 2011; Hampson et al. 2006). However, differences in classification accuracy between males and females have been found only during the display of subtle emotional expressions (Hoffmann et al. 2010; Montagne et al. 2005). Gender has also been shown to mediate response tendencies. Although the effect on explicit behavioral tendencies has largely been neglected, implicit paradigms have shown that women exhibit increased backward movement, as measured by COP displacement, in response to unpleasant stimuli as well as greater defensive reactivity to aversive pictures, as measured by a deceleration in heart rate. Men, in contrast, exhibit increased anterior movement in response to unpleasant stimuli and less defensive reactivity (Bradley et al. 2001; Hillman et al. 2004).

In this vein, it is important to implement more ecologically valid paradigms that investigate such phenomena reliably. So far, there have been no systematic investigations of whether emotion perception and response behavior are, indeed, modified depending on whether stimuli are presented in the “pictorial” space of screens or in a “visual” space shared with other agents that achieves a feeling of co-presence. A virtual reality (VR) paradigm presents a suitable method to investigate emotion perception, perceived emotional intensity, and the associated action tendencies under different conditions (Seidel et al. 2010b; Visch et al. 2010).

### The research aim

The present study follows two central goals: by taking a multisystem approach, it aims to shed light on how different virtual environments affect the perception of emotional interactions (i.e., anger, sadness, affection, happiness) and trigger action tendencies in response to emotional interactions. More specifically, we tested whether co-presence modulates perception and associated response behavior toward emotional body language by creating two display conditions within a virtual reality: a pictorial display condition that simulates a conventional experiment conducted on a computer monitor and a visual display condition containing valid sensorimotor contingencies that enhance the participant’s sense of co-presence and agency (for the conceptual distinction between pictorial and visual spaces, see Koenderink and van Doorn 2012; Troje 2019). We then compared the explicit rating of emotional valence as well as the explicit and implicit tendency to act in the two display conditions. Implementing these measures, we cannot only evaluate a possible influence of the virtual condition on each of them but we can also further elucidate the linkage between perception and behavioral action. Thus, we asked participants to observe emotional interactions within the two conditions and explicitly evaluate (a) the behavioral tendency to approach or avoid the observed scene, and (b) the emotional valence (i.e., positive or negative) of the scene. These explicit ratings reflect a conscious cognitive judgment of the observed stimulus. Moreover, we measured implicit bodily response behavior toward the stimulus via COP displacement, allowing us to unmask automatic response behavior that takes place at earlier stages of emotion perception processing. To assess whether subjective presence was associated with autonomic arousal, we applied a continuous skin conductance measure.

Finally, we explored whether and how emotion perception and response tendencies were modulated by the observer’s gender. More specifically, we asked whether women and men differed in judging emotional valence and in their bodily response to emotional interactions both explicitly and implicitly.

## Materials and methods

### Participants

A total of 76 healthy adults, including 39 women (*M*_age_ = 23.21, SD = 2.58) and 37 men (*M*_age_ = 25.86, SD = 4.64) with normal or corrected-to-normal vision participated in the experiment. All participants gave written informed consent to take part in this study. None of the participants reported any history of psychiatric or neurological disorders and no current abuse of drugs or any psychoactive medication. The protocol was approved by the Institutional Ethics Committee and was conducted according to the principles of the Declaration of Helsinki.

We assessed aspects of personality with the emotional competence questionnaire (EKF, Rindermann 2009) and controlled for each participant’s affective state with Beck’s Depression Inventory (BDI-II; Beck et al. 1996) and the State-Trait Anxiety Inventory (STAI; Spielberger et al. 1983). With regard to state anxiety, participants’ average scores ranged from 23 to 57 (*M*_female_ = 34.56; SD_female_ = 7.56; *M*_male_ = 35.62, SD_male_ = 8.01). Scores on the trait anxiety questionnaire ranged from 21 to 59 (*M*_female_ = 37.10; SD_female_ = 9.18; *M*_male_ = 35.73, SD_male_ = 8.42), with higher scores indicating greater anxiety. BDI scores ranged from 0 to 22 (*M*_female_ = 5.68; SD_female_ = 5.18; *M*_male_ = 4.38, SD_male_ = 3.65). Female and male participants did not differ in their mean STAI or BDI-II scores as shown by nonsignificant Mann–Whitney *U* tests (all *p*s > 0.05, see supplementary material S1). Furthermore, no correlation was found with our dependent variables, see supplementary material table S2–S4.

### Stimuli

#### Creating the stimulus set

Stimuli were created with a motion capture system (VICON, Oxford, UK) that recorded the position of 41 markers attached to predefined anatomical landmarks. Eight pairs of nonprofessional actors were asked to portray one of the following four emotional states within a dialog: anger, sadness, affection, or happiness. Due to its strong interpersonal component, we included affection even though it is not considered to be a basic emotion (see Clarke et al. 2005). To increase the variability of the movements, each emotional state was portrayed in three intensities: low, medium and high. Actors were specifically asked to act out the same emotion. To facilitate a symmetric behavioral pattern, all actors received scripts of emotional situations that they were instructed to perform. They were asked to act intuitively within the context of the given situation, allowing for freedom in their expressions. In the next step, we postprocessed the motion capture data and edited the scenes into 3-s interaction sequences.

#### Stimulus selection

Finally, we randomly selected 96 point-light stimuli (24 × 4 emotions) and tested their recognizability in a separate pilot study (*n* = 36). Scenes had to meet two main criteria: first, mean valence ratings had to reflect the displayed emotion (i.e., negative ratings for anger and sadness and positive ratings for affection and happiness). Second, mean valence ratings between − 1 and 1 were excluded due to their ambiguity or lack of emotionality. Finally, the remaining trails were balanced (i.e., 12 sequences per emotion), leading to a final stimulus set of 48 emotional scenes. For the present study, we applied a MoSh algorithm (Loper et al. 2014) to the motion capture data (i.e., point-light displays) to create avatars for the virtual environment (for exemplary movies, see supplementary material Video S1–S8).

### Materials and apparatus

#### Virtual environment

**Fig. 1:**
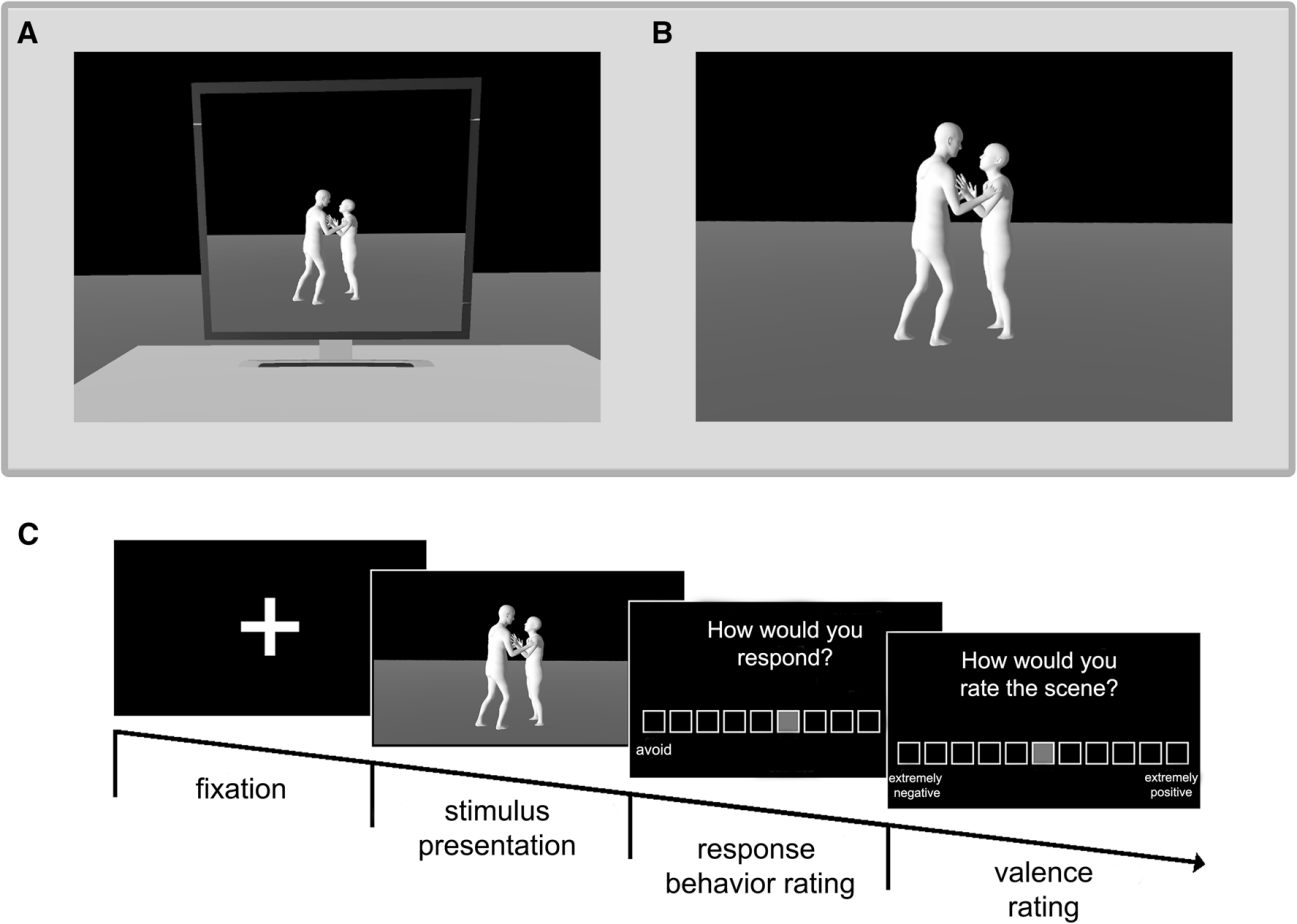
Display conditions and experimental timeline. The stimulus sequences are presented within **a** the pictorial condition (i.e., a virtual computer monitor) and **b** the visual condition (i.e., the observer shares a common space with the stimuli). **c** Temporal structure of one trial

Using the Unity3D game engine by Unity Technologies (http://unity3d.com), we created two display conditions that we presented to participants in VR. The visual condition depicts a three-dimensional space in which participants share a common space with the dynamic avatars who are engaging in social interaction. The visual scene is presented stereoscopically and also responds with motion parallax contingent to the participant’s body movements. In the pictorial condition, participants are standing in front of a virtual computer screen placed on a virtual table. In this case, stereopsis and motion parallax indicate to the participant that the screen is flat and that the visual scene being presented is projected onto it. Therefore, it is thought to elicit a lower subjective feeling of co-presence than in the visual condition (see Fig. 1a, b). Using the HTC Vive Headset, the stimulus material was presented via SteamVR software (http://steamvr.com). In both cases, VR was rendered at 90 Hz with a display resolution of 2160 × 1200 pixels and a field of view of about 110°. Stimuli were presented in front of the participants who were placed at the same location at the beginning of each sequence.

#### Explicit ratings

Rating responses were collected with the HTC Vive controller placed in the participant’s dominant hand. After stimulus presentation, participants were shown a virtual rating scale and asked to judge (a) their tendency to respond to the stimulus (i.e., whether they would like to approach or avoid the scene) and (b) the valence of the stimulus (i.e., how positively or negatively they perceived it).

#### Implicit measurements

Participants stood on a mobile force plate (Accu Gait System, AMTI Force and Motion, Watertown, MA) throughout the experiment. Changes in force and torque in the *x*, *y*, and *z* dimensions were recorded during each stimulus presentation (i.e., for 4 s at a rate of 50 Hz). Continuous EDA measurement was applied to control for sympathetic nervous activity. We did this by tying two Ag–AgCl electrodes filled with an electrolytic gel mixture (GEL101) to the distal phalanges of the nondominant hand. The signal was recorded at a sampling rate of 1000 Hz. Physiological responses were registered through a physiological amplifier BIOPAC MP36 (BIOPAC Systems, Inc., Goleta, CA).

### Experimental design

In the present study, we implemented a crossover design in which all participants underwent both conditions (i.e. visual condition, pictorial condition). The conditions were separated into two blocks. The first block that was presented was alternated between participants. More specifically, if one participant started with the visual condition, the next participant would start with the pictorial condition. This was done to prevent a systematic error that may occur due to exhaustion or habituation.

### Procedure

Prior to the actual experiment, participants were asked to fill out a self-administered battery of questionnaires assessing emotional competencies and personality aspects (see Participants section above). The experiment started with a 2-min baseline recording of the EDA while participants looked at a gray screen while standing on the force plate. Next, they were instructed to carry out a test version of the experiment to familiarize themselves with the task. During the test version, emotional sequences appeared in the same order for all participants. These sequences were not shown in the main experiment. Subsequently, the actual experiment started. While standing on a force plate, two blocks of trials were shown, each containing all 48 emotional interactions presented in a pseudorandomized order. In one of the blocks, the trials were presented on a virtual screen (pictorial condition, Fig. 1a) and in the other they were presented in the virtual, open 3D space (visual condition, Fig. 1b).

Force plate data (i.e., implicit response behavior) were collected during the presentation of a sequence. Following each stimulus presentation, participants were instructed to make two explicit judgments (see Fig. 1c): first, they explicitly indicated their preferred behavioral response (i.e., explicit response behavior). More specifically, they were asked to observe the interactive avatars while standing in front of them and explicitly rate their tendency to approach or avoid the avatars displaying their particular emotional body expressions on an 11-point scale ranging from − 5 (avoid) to + 5 (approach) with 0 (neither) marking the center of the scale. Second, participants were asked to judge the valence of the interaction on a 11-point scale ranging from − 5 (extremely negative) to + 5 (extremely positive) with 0 (neutral) marking the center of the scale. After one half of each block, as well as at the end of each block, participants were asked to indicate how present they felt within the scene (‘How present did you feel within the scene?’) using a 7-point scale ranging from 1 (not at all) to 7 (extremely present).

### Data analysis and statistics

#### Electrodermal activity

In the first step, the EDA signal was downsampled to 10 Hz and filtered using a fourth-order Butterworth low-pass filter with a cutoff frequency of 5 Hz. Afterwards, the signal was smoothed using a first-pass boxcar and second-pass Parzen window with a length of 150 samples. Skin conductance response (SCR) peaks were then determined according to Kim et al. (2004) and refined using a forward-ascending nearest-maximum search. An SCR was accepted as valid when above 0.02 µS and below 1 µS. To extract all SCRs, phasic component analysis was conducted using a trough-to-peak analysis within a 1–6 s delayed window after stimulus onset (Dawson et al. 2017). In the next step, a square-root transformation was applied to normalize the data. Finally, we calculated mean values for each emotional category (anger, sadness, affection, happiness) per display condition (pictorial, visual).

#### Center of pressure displacement

Force plate data were preprocessed and analyzed using MATLAB 2018a (MathWorks, Inc., Natick, MA). In the first step, data were low-pass filtered with a cutoff frequency of 8 Hz. For each trial, we calculated the mean COP displacement in the anteroposterior direction (COP-AP, in mm, corresponding to the COP position during the stimulus presentation relative to the COP position at the first frame of the stimulus). Next, we identified outliers and excluded trials from further analysis when the range of mediolateral COP displacement exceeded 8 cm, indicating movement of the feet. Finally, we calculated the postural responses for each emotional sequence within both display conditions per participant.

#### Presence

All ratings were preprocessed using MATLAB 2018a (MathWorks, Inc., Natick, MA). Statistical analyses were performed with IBM SPSS Statistics (version 25, IBM Cor, Armonk, NY). Mean values of subjective presence were calculated per participant and per display condition (i.e., pictorial, visual). A Wilcoxon signed-rank test was then conducted per group (i.e., males and females) to compare presence ratings within the pictorial and the visual condition. Following Rosenthal (1994), effect sizes *r* were calculated as *Z* statistic divided by square root of the sample size *N* with *N* being the number of total observations. To assess whether greater subjective presence was associated with higher autonomic arousal, we calculated Spearman correlation coefficients between subjective presence and skin conductance responses toward the emotional scenes.

#### Explicit and implicit measurements

We tested the influence of display condition and emotional category on the following dependent variables: (1) valence judgment, (2) explicit response behavior, (3) implicit response behavior. More specifically, three separate 2 (display condition: visual, pictorial) × 4 (emotion: anger, happiness, affection, sadness) × 2 (gender: male, female) repeated measures ANOVAs with the between-subject factor “participant gender” were conducted. With respect to implicit response behavior, we used COP mean displacement in the anterior–posterior direction as the dependent variable.

#### Relationship between valence judgment and response behavior

Finally, we aimed to explore whether the perceived valence of an emotional scene is correlated with the tendency to respond with approach or avoidance. To do so, we calculated correlation coefficients per participant for explicit judgments—that is, reported valence (positive/negative) and response behavior (avoid/approach). We did the same for valence judgment and implicit response behavior (COP displacement). In the next step, we applied a Fisher’s *Z* transformation. We conducted one-sample *t* tests per group (male, female) and for each condition (pictorial, visual) to analyze whether the correlation was significant. Last, we conducted an independent-samples *t* test of the mean correlations of women versus men.

## Results

### Subjective presence

**Fig. 2:**
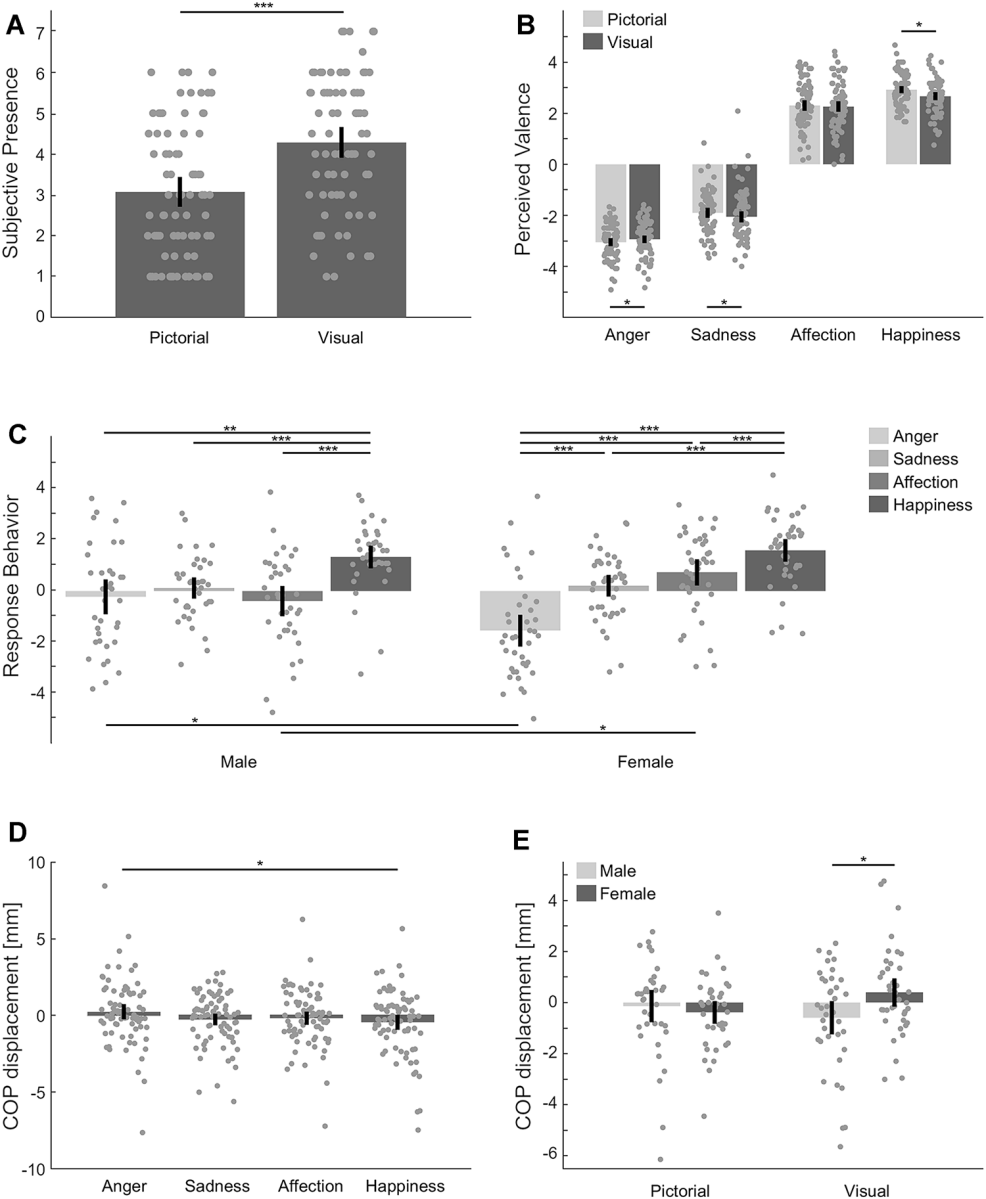
Bars (including their standard errors and individual data points) showing **a** the mean subjective presence ratings per display condition (i.e., pictorial, visual) and **b** mean valence ratings per emotion separated by display condition (pictorial, visual) Only significant differences between the conditions are indicated. Post hoc tests indicated that within both conditions, all emotions differed significantly from each other (all *p*s < .05). **c** Mean explicit response behavior ratings for each emotion per group (i.e. male, female); note that significance bars above indicate differences between emotions for each gender, whereas significance bars below indicate differences between men and women for each emotion. **d** Mean COP displacement in anterior (i.e., positive values) and posterior (i.e., negative values) direction per emotion. **e** Mean COP displacement in anterior–posterior direction for each condition, separated by gender. Only significant differences between men and women are indicated. For men, COP displacement did not differ between the pictorial and visual condition (*p* > 0.05), whereas for women, COP displacement differed significantly between conditions (*p* < 0.05). Significance level is indicated by asterisks (*p < 0.05; ***p < 0.001)

First, we evaluated whether the perceived subjective presence differed between the two display conditions. Descriptive statistics revealed higher mean values of subjective presence in the visual condition (*M* = 4.28, SD = 1.65) compared to the pictorial condition (*M* = 3.07, SD = 1.60), see Fig. 2a. We applied a Wilcoxon signed-rank test separately for males and females. Data showed that both males (*Z* = − 4.98, *p* < 0.001, *r* = 0.58) and females (*Z* = − 4.59, *p* < 0.001, *r* = 0.52) felt significantly more present in the visual condition, confirming that subjective experience differed between the two conditions. To investigate whether increased subjective presence resulted in increased physiological responses toward the emotional sequences, we calculated Spearman correlation coefficients between the mean phasic electrodermal response and subjective presence, both per display condition (pictorial, visual). We found no significant correlations (all *p*s > 0.05), indicating that the subjective presence did not relate to the physiological responses elicited within either of the display conditions.

### Does the display condition influence emotional valence perception?

In the next step, we calculated a 2 (display condition: visual, pictorial) × 4 (emotion: anger, sadness, affection, happiness) × 2 (gender: male, female) repeated measures ANOVA with valence as a dependent variable and gender as a between-subject factor. We found a main effect of display condition, *F*(1, 74) = 6.94, *p* = 0.01, $\eta_{{\text{p}}}^{2}$ = 0.09, indicating that, overall, stimuli were perceived slightly more negatively in the visual compared to the pictorial condition (*M*_diff_ = − 0.09,* p* = 0.01)*.* Furthermore, we found a main effect of emotion, *F*(1, 74) = 1174.43, *p* < 0.001, $\eta_{{\text{p}}}^{2}$ = 0.94. Post hoc analyses showed that all valence ratings differed significantly from each other (all *p*s < 0.001). As expected, descriptive statistics indicated negative mean valence ratings for anger and sadness, whereas affection and happiness sequences, on average, were rated positively, see Fig. 2b.

Further, we found a significant interaction between display condition and emotion category, *F*(3, 222) = 7.76, *p* < 0.001, $\eta_{{\text{p}}}^{2}$ = 0.10, indicating that the display condition did not affect valence perception of all emotions equally. Post hoc analyses revealed that anger (*M*_diff_ = − 0.12,* p* = 0.02) was perceived slightly more negatively and happiness (*M*_diff_ = 0.26,* p* < 0.01) slightly more positively within the pictorial condition, whereas sadness was perceived more negatively within the visual condition (*M*_diff_ = 0.16, *p* = 0.01), see Fig. 2b. Overall, after Bonferroni corrections, these effects were only marginal. Affection was not perceived differently between display conditions (*p* > 0.05). This interaction implies that the observed main effect of viewing condition is mainly carried by the sequences that are displaying sadness and happiness. With regard to gender effects, mean valence ratings of each emotion did not differ between women and men, *F*(3, 222) = 0.06, *p* > 0.05, $\eta_{{\text{p}}}^{2}$ = 0.001. Moreover, the interaction between display condition and gender was not significant, *F*(1, 74) = 0.04, *p* > 0.05, $\eta_{{\text{p}}}^{2}$ = 0.10, indicating that the display condition did not affect valence perception of women and men differentially.

### Does the display condition influence explicit response tendencies?

To explore the effects of the display condition and emotion category on reported response behavior, we calculated a 2 (display condition: visual, pictorial) × 4 (emotion: anger, sadness, affection, happiness) × 2 (gender: male, female) repeated measures ANOVA with explicit response behavior rating as a dependent variable and gender as a between-subject factor. We found no mediation by the display condition for this analysis as reflected by a nonsignificant main effect, *F*(1, 74) = 0.64, *p* > 0.05, $\eta_{{\text{p}}}^{2}$ = 0.01. We found a main effect of emotion, *F*(3, 222) = 29.26, *p* < 0.001, $\eta_{{\text{p}}}^{2}$ = 0.28, indicating that anger elicited greater avoidance tendencies than all other emotions (all *p*s < 0.05). In contrast, happiness elicited greater approach tendencies than all other emotions (all *p*s < 0.001). Sadness and affection did not elicit differential response tendencies (*p* > 0.05).

Furthermore, we found an interaction with the gender of a person (*F*(3, 222) = 8.17, *p* < 0.001, $\eta_{{\text{p}}}^{2}$ = 0.09). Post hoc analyses showed that for men, happiness elicited positive and significantly higher approach tendencies than all other emotions (all *p*s < 0.01). For women, we found the same effect as well as negative and significantly higher avoidance tendencies for anger sequences as compared to all other emotions (all *p*s < 0.001), see Fig. 2c.

Testing for gender-specific response tendencies with respect to different emotion categories, we found greater avoidance tendencies in response to anger sequences [*M*_diff_ = − 1.32*, p* < 0.01, 95% CI (− 2.24, − 0.41)] as well as higher approach tendencies toward affection sequences in women as compared to men [*M*_diff_ = 1.13, *p* < 0.01, 95% CI (0.35, 1.92)], see also Fig. 2c. The display condition in which emotional interactions were presented, however, did not exert differential effects on men and women as reflected by a nonsignificant interaction, *F*(1, 74) = 0.32, *p* > 0.05, $\eta_{{\text{p}}}^{2}$ = 0.004.

### Does the display condition affect implicit response tendencies?

To explore the effect of the display condition on automatic reaction tendencies toward the emotional scenes, we calculated a 2 (display condition: visual, pictorial) × 4 (emotion: anger, sadness, affection, happiness) × 2 (gender: male, female) repeated measures ANOVA with COP displacement in anterior–posterior direction as a dependent variable and gender as a between-subject factor. Results revealed no main effect of the display condition [*F*(1, 74) = 0.61, *p* = 0.44, $\eta_{{\text{p}}}^{2}$ = 0.01], but a main effect of emotion [*F*(3, 222) = 3.05, *p* < 0.05, $\eta_{{\text{p}}}^{2}$ = 0.04, see Fig. 2e]. More specifically, anger elicited significantly higher anterior movement than happiness scenes [*M*_diff_ = 0.69 mm, *p* < 0.05, 95% CI (0.004, 1.37)]. This effect was not mediated by a person’s gender [*F*(3, 222) = 0.98, *p* = 0.40, $\eta_{{\text{p}}}^{2}$ = 0.01]. However, we did find an interaction effect between display condition and gender [*F*(1, 74) = 8.54, *p* < 0.01, $\eta_{{\text{p}}}^{2}$ = 0.10]. Post hoc analyses showed that women and men, on average, presented implicit avoidance behavior (i.e., increased posterior movement) in the pictorial condition. However, women displayed increased anterior movement within the visual condition, indicating approach behavior, whereas men, on average, displayed posterior movement (*M*_diff_ = 0.99 mm*, p* < 0.05, 95% CI (0.14, 1.85)], see Fig. 2f.

### Is there a link between valence judgment and response behavior?

**Fig. 3:**
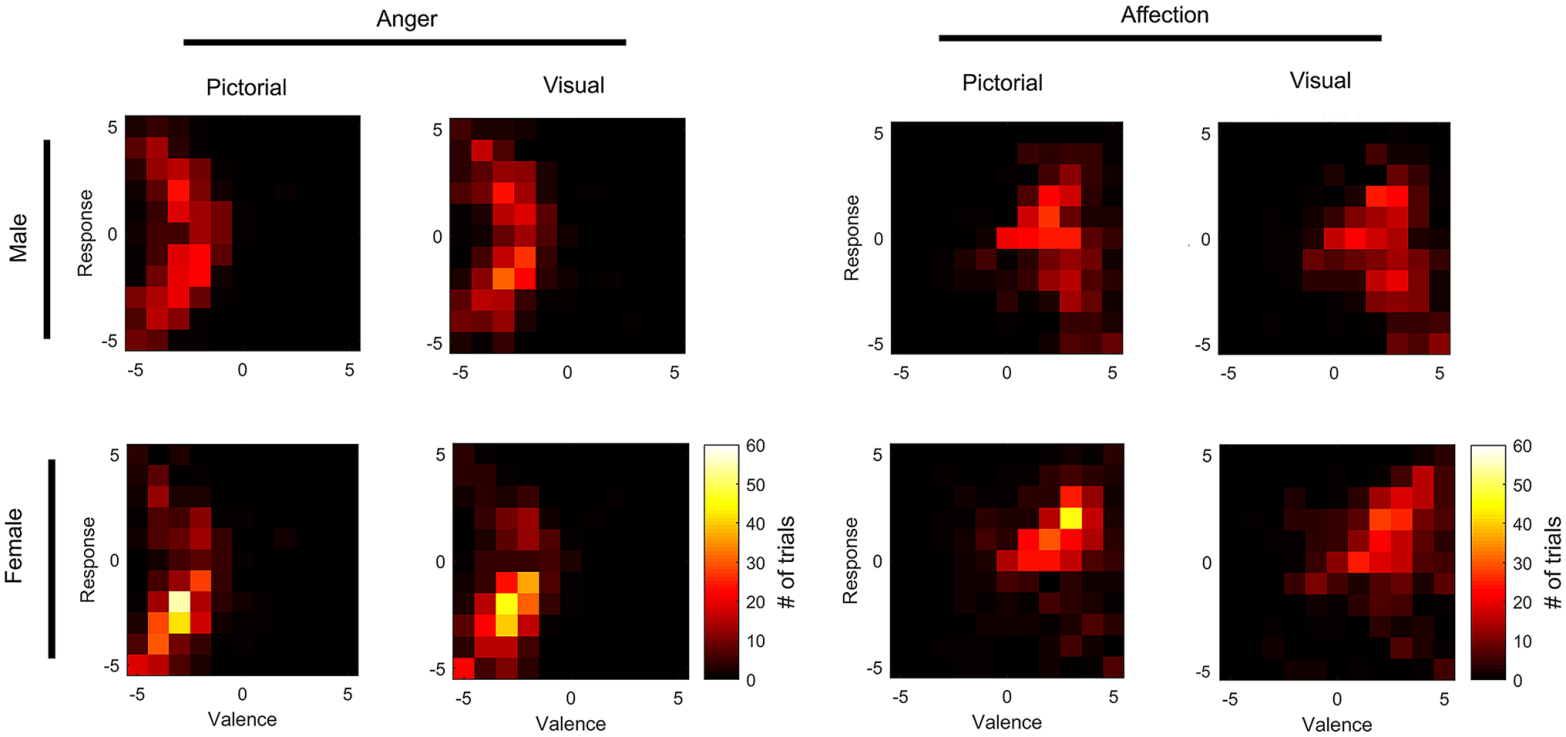
Distribution of trials indicating how often specific valence × response behavior rating combinations were observed. All heat maps separated for **a** anger and **b** affection sequences in each space (pictorial, visual) and gender (female, male)

Due to the gender-mediated discrepancies between valence judgment and explicit response tendencies we observed in anger and affection sequences (compare Fig. 2b, c), we explored the phenomenon in more detail. Although men tended to judge anger sequences just as negatively as women did, about half of all male participants (i.e., 51.35% in the pictorial condition and 48.65% within the visual condition) indicated that they would like to approach the situation. Figure 3 illustrates how often specific valence and (explicit) response behavior rating combinations were observed for anger and affection sequences as well as within each condition (i.e., pictorial and visual).

For each participant as well as for both display conditions, we calculated correlation coefficients between explicit valence and response behavior ratings. With respect to anger sequences, correlation coefficients did not differ significantly from zero in either condition for the male population (all *p*s > 0.05). For women, the Bonferroni-corrected one-sample *t* tests indicated that correlation coefficients differed significantly from zero within the pictorial [*M* = 0.42, *t*(37) = 3.34, *p* < 0.01, *d* = 0.54, 95% CI (0.17, 0.68)], and the visual condition [*M* = 0.36, *t*(37) = 2.75,* p* < 0.05, *d* = 0.45, 95% CI (0.09, 0.62)].

We found the same effect for affection sequences. Whereas correlation coefficients found for men did not differ significantly from zero in either condition (all *p*s > 0.05), Bonferroni-corrected one-sample *t* tests indicated that correlation coefficients for women differed significantly from zero within both the pictorial [*M* = 0.44, *t*(38) = 3.97, *p* < 0.01, *d* = 0.64, 95% CI (0.21, 0.66)], and the visual condition [*M* = 0.38, *t*(36) = 3.29, *p* < 0.01, *d* = 0.54, 95% CI (0.15, 0.61)]. Although women and men did not seem to differ in their judgment of emotional valence, there was a congruent relationship with reported response tendencies for women (i.e., avoidance in response to anger and approach in response to affection), whereas there seemed to be no relationship between these variables for men. Moreover, Bonferroni-corrected independent-samples *t* tests indicated that mean correlations between valence judgment and explicit response behavior rating for anger sequences differed significantly between women and men in the pictorial condition [*M* = − 0.58, *t*(72) = − 3.12, *p* < 0.05, *d* = 0.73, 95% CI (− 0.95, − 0.21)], but not in the visual condition [*t*(73) = − 2.31, *p* = 0.10]. For affection, mean correlations between women and men did not differ in either of the display conditions [pictorial: *t*(74) = − 2.45, *p* = 0.07, visual: *t*(72) = − 2.32, *p* = 0.09].

With respect to valence judgment and implicit response tendencies (i.e., COP displacement), results showed that correlations were not significant for either women or men, indicating that there was no relationship between the explicit valence judgment and the automatic tendency to avoid or approach (all *p*s > 0.05).

## Discussion

Using a multisystem approach, we aimed to shed light on how different virtual environments influence the perception of emotional body language and associated action tendencies. We did this by comparing two virtual display conditions: a pair of interacting partners was presented within either a pictorial condition in which participants viewed the emotional stimuli depicted on a screen or a visual condition in which participants shared the same virtual room with the interactive partners. We asked whether display conditions would affect explicit judgments (i.e., valence perception and response behavior) as well as implicit response behavior differentially. Overall, our results indicate that the visual condition in which the observer is co-present with other (virtual) agents elicited a stronger feeling of presence in the virtual world as indicated by subjective ratings. However, the display condition exerted only marginal effects on valence judgments and no effects on explicit response behavior. Implicit response behavior (i.e., COP displacement) was affected differentially by the display conditions—however, in a gender-specific manner. Our results showed that valence perception (i.e., the impression of how positively or negatively a scene is perceived) does not differ between women and men. In contrast, gender seems to mediate how individuals respond toward a given stimulus—both explicitly and implicitly. In the following sections, we shall discuss these findings in greater detail.

### Valence perception

With respect to valence perception, the display condition exerted only marginal effects on how positively or negatively an emotional interaction was perceived depending on the emotional category presented. More specifically, anger and happiness were judged to be more intense in the pictorial condition, meaning that anger was perceived more negatively and happiness was perceived more positively. Sadness, in contrast, was judged more negatively in the visual condition. Affection was not perceived differently between display conditions.

Prior studies have demonstrated that immersive virtual environments (i.e., an environment in which the participant is in “presence mode”) intensify the emotional response of the viewer as measured by the subjective experience of arousal. This may lead, in turn, to an intensified perception of the content being viewed (Estupiñán et al. 2014; Visch et al. 2010). However, our data revealed that this is not necessarily the case. Instead, our results point toward a differential effect: sadness was judged to be only slightly negative, whereas anger and happiness were judged to contain greater emotional valence. Whereas sadness sequences were rated to contain less emotional valence, happiness and anger were rated as being more intense. Thus, whether a stimulus is perceived more or less intensely within a more immersive environment may depend on its valence intensity. Slater (2009) has discussed this phenomenon in terms of realistic physiological, emotional, and behavioral responses due to place illusion (i.e., a sense of being present in the virtual environment despite knowing at a higher cognitive level that the situation is not real). According to his view, more immersive environments may change our conscious cognitive perception into a more realistic, instead of a generally more intense experience. Note that valence judgments, overall, differed only slightly between the pictorial and the visual condition in our study, suggesting that the conscious evaluation of an observed emotional interaction does not seem to be influenced that strongly by the display condition. This lack of a relevant difference in valence perception is also in line with the results of a pilot study carried out by Estupiñán et al. (2014), who found that mean valence values for images of human concerns (i.e., scenes violating human rights) displayed within a VR head-mounted display did not differ significantly from the reference of the Geneva Affective Picture Database (GAPED) displayed on a computer screen.

Turning to the lack of relevant differences in valence judgements, it has to be noted that the increased sense of subjective presence was not accompanied by increased physiological arousal (i.e., increased skin conductance response) in the present study. However, previous studies have proposed that greater presence evokes greater autonomic responses (e.g., increased heart rate measures and, to a lesser degree, increased skin conductance response; see Meehan et al. 2002), especially when participants were exposed to stressful virtual environments. Freeman et al. (2005) pointed out that presence and emotion perception are related only for arousing stimuli, and that increased arousal can lead to an intensified perception of the emotional content being viewed. Hence, it seems plausible that the perception of emotional valence within highly immersive environments may be altered especially when stressful or highly arousing stimuli are displayed (Diemer et al. 2015; Freeman et al. 2005; Visch et al. 2010). In the present study, however, the stimulus material was not created to induce stress or other emotional distress in the observer, but rather to reflect real-world emotional interactions that would allow us to investigate the perception of affective states in others. Thus, it may be possible that changes in valence perception between the display conditions may occur when highly arousing stimuli are used.

Turning to the influence of gender on valence perception, we found that women and men did not differ. Our data indicate no gender differences in valence judgments for either more intense emotions such as happiness and anger or less intense emotions such as sadness and affection. Although many studies have suggested a female superiority in emotion recognition (e.g., Alaerts et al. 2011; Hall and Matsumoto 2004; Hampson et al. 2006), other studies have found that gender effects occur only when highly subtle emotions are presented compared to more intense, prototypical emotional displays (Hoffmann et al. 2010; Montagne et al. 2005). The lack of agreement on gender differences between the current and prior studies may be due to methodological differences. Whereas most studies assessed performance (i.e., accuracy) or reaction times when assigning emotional displays to their respective category (e.g., anger, happiness), we assessed how strongly the valence of a given stimulus is perceived for different emotional displays (Alaerts et al. 2011; Hampson et al. 2006) and, therefore, focused on a more subjective dimension of perceiving emotional body language.

### Response behavior

#### Explicit response behavior

We found that explicit response behavior is not influenced differentially by the display condition, meaning that the urge to approach or avoid an emotional scene is virtually the same. Similar to valence judgments, explicit response behavior is thought to represent complex cognitive evaluations (in contrast to automatic response behavior) that may be shaped by individual factors, such as personality traits. Although we can only speculate about why co-presence does not affect explicit response tendencies, it seems plausible that participants reflected actively on how they would react if this scenario were to occur in a “real-life” situation. Applying such a strategy could certainly dampen the experience and break the illusion of being inside the virtual world despite knowing that it is not real (Slater 2009). As a result, the cognitive evaluation to avoid or approach the situation will not be affected differently by virtual co-presence.

Interestingly, we found that explicit response behavior is mediated by the observer’s gender. Whereas women tend to report greater avoidance tendencies toward anger than men, they also report greater approach tendencies toward affection than men. Explicit response tendencies represent cognitive evaluations of a stimulus which may be affected by social learning and reinforcement. It is assumed that men learn to hide helplessness and express aggression related emotions such as anger more openly, whereas women are reinforced when showing helplessness and suppressing anger (Fischer 1993). Thus, with respect to anger sequences, it seems plausible that women tend to respond with avoidance while men respond with approach. However, there are notable within group inconsistencies in the tendency to avoid or approach. Thus, the impact of different personality traits on helping behavior e.g., altruism (Wang and Wang 2008) and agreeableness (Graziano et al. 2007), might further elucidate those inconsistencies. Moreover, Seidel et al. (2010b) suggest that the gender of the interacting agents might also be an influencing factor on conscious as well as automatic behavioral tendencies. The authors suggest that social learning possibly turned male faces expressing negative emotions to salient social cues, which communicate the message to respond with avoidance.

#### Implicit response behavior

Whereas explicit response behavior has been largely neglected in the literature*,* implicit response behavior has been studied quite extensively (e.g., Bradley et al. 2001; Hillman et al. 2004; Horslen and Carpenter 2011; Seidel et al. 2010b). We found that automatic response tendencies depend on the emotion being displayed. On average, anger elicited greater forward movement than happiness. Although this may initially seem counterintuitive, because anger has also been shown to elicit avoidance tendencies (e.g., Chen and Bargh 1999; Marsh et al. 2005), other studies suggest that humans can be motivated to approach, confront, and overcome the social challenge posed by angry expressions (Carver and Harmon-Jones 2009; Wilkowski and Meier 2010). This is in line with findings by Stins and Beek (2007) who reported that unpleasant images elicit a forward COP displacement (of about 1 mm). It is noteworthy, however, that we observed high variances in the implicit behavior to approach or avoid. This indicates that other factors such as learning experiences or certain personality factors may play a mediating role in the behavioral tendency to respond.

Moreover, we found an interaction between the display condition and the gender of a person when it came to implicit responses. Whereas women and men generally tended to display backward movements in the pictorial condition, women displayed approach tendencies in the visual condition. This is an interesting finding, because it indicates that the display condition may indeed differentially affect automatic responses toward emotional stimuli—at least in women.

However, it should be pointed out that, overall, the mean displacement is extremely small (less than 1 mm) and should therefore not be overinterpreted. Interestingly this “lack” of implicit behavioral tendencies toward emotional displays has also been reported by Seidel et al. (2010a), who showed that only depressive patients—but not healthy controls—displayed implicit avoidance behavior toward angry faces. Moreover, healthy controls also did not display significant behavioral tendencies toward happy faces. This is in line with findings by Stins and Beek (2007) reporting that viewing emotion eliciting pictures had little effect on body sway. The authors suggested that passive viewing might be coupled only weakly to posture, and that an effective way to probe the emotion-posture system would be to induce emotional states that are relevant for postural control, such as anxiety.

#### The influence of perspective

With respect to both, implicit and explicit response tendencies, an important aspect that should be highlighted is the influence of viewing perspective. Within both conditions, participants remain in an observing position, or third-person perspective, in which the observed actions are not directed at the viewer. That means, the viewer passively observes the actors' interaction. An actor, or first-person perspective, in contrast, is created when the viewer is transported into the perspective of an actor. Research shows that spatial presence is increased in the actor perspective as compared to the observer perspective (van den Boom et al. 2015). Although speculative, it may be possible that a condition in which the viewer is put in an actor perspective, in which the observed actions are directed towards the participant, stronger approach- or avoidance tendencies could be observed.

### The link between valence and explicit response behavior

Finally, we conducted an exploratory analysis of gender-mediated discrepancies between valence judgment and associated response tendencies toward anger and affection sequences. Although some studies have reported that negatively valenced stimuli such as angry faces signal the request to go away (i.e., avoidance), others have demonstrated that a negative evaluation of stimuli does not necessarily evoke avoidance behavior (Carver and Harmon-Jones 2009; Horstmann 2003; Seidel et al. 2010b; Wilkowski and Meier 2010). Wilkowski and Meier (2010), for instance, have argued that angry facial expressions communicate the intention to confront a person aggressively. Thus, they pose an important social challenge that should predispose individuals to engage in approach-motivated behavior to confront or overcome them.

Our data showed that while there is a positive link between valence judgment and explicit response tendency toward anger in women (i.e., negative valence judgment and the behavioral tendency to avoid), this link is absent in men. This is due to a high variability in explicit response behavior toward anger within the male population. Whereas some participants indicated they would like to approach the situation, others reported feeling the tendency to withdraw. Although a person’s gender does seem to play a role in the relationship between valence and explicit response behavior for anger, it is not the sole contributor. Personality models, for instance, suggest that certain traits foster or inhibit response behavior (see Carver 2005, for a review). We consider these to be interesting results, because they highlight the complexity of the phenomenon.

## Conclusion

The present study showed that virtual environments in which the observer shares a common space with virtual agents increase the observer’s subjectively reported presence when observing emotional interactions. It further showed that the cognitive evaluation of stimulus valence is largely independent of the display condition (i.e., visual vs. pictorial) and that it does not differ between women and men. These findings support the notion that humans are highly adept at recognizing emotional stimuli from emotional body language, even within more abstract settings such as pictorial designs.

Moreover, we did not observe differential effects of the display condition on explicit response behavior. However, we did observe a gender-mediated effect of display condition on implicit response behavior. Although the mechanism is still unclear, it seems that being inside a “visual” space may act more strongly on automatic motivational tendencies but not on higher level cognitive evaluations. We speculated that this might be due to a break in place illusion caused by active cognition evaluation of the scene.

Interestingly, a person’s gender also mediated explicit response behavior. Women indicated a tendency to explicitly avoid anger scenes and approach affection scenes more strongly than men, suggesting that they react with a greater defensive motivation toward anger as well as greater appetitive motivation toward affection than men. Interindividual differences in explicit rating behavior indicated that a proportion of the male participants explicitly wanted to approach anger scenes. Perhaps this was due to a motivation to confront or overcome a social challenge posed by angry expressions (Carver and Harmon-Jones 2009; Wilkowski and Meier 2010). Moreover, we found that the display condition did not affect men and women differently with respect to valence or explicit response behavior ratings.

Finally, we discovered a closed link between valence perception and congruent explicit response behavior in women. However, this link was absent in men.

## Limitations and future implications

Virtual reality offers many options as well as challenges. Whereas it can serve as a way to manipulate experimental settings in a highly controlled manner, its complexity and the effects it has on human perception are not well understood. Here, we applied a virtual reality paradigm comparing a “pictorial” condition (i.e., a space that simulates a computer experiment on a computer screen) to a “visual” condition (i.e., a condition with a shared space between the observer and virtual agents). Although this allowed us to modify the subjective experience of presence within the virtual environment, we did not exhaust its full potential. More specifically, while the difference in subjective presence can be assessed quantitatively, it remains open whether it is a meaningful difference. Across both conditions, the viewer remains in an observer perspective and is not able to experience the scene from an actor perspective or influence the course of the emotional interaction. Recent studies have proposed that the environment is thought to be understood and perceived through our interaction with it. In other words, perceptual experience is no longer thought to result from passive information processing, but is “enacted” via regulation of sensorimotor loops and active exploration of the environment (Engel et al. 2013; Froese et al. 2014). Thus, future studies should tackle the role of an actor perspective, potentially within an interactive VR setting. In this regard, response behavior tendencies, such as approach and avoidance, can be measured by instructing the participant to physically walk towards (approach) or away from a scene (avoid). Thereby, the rating continuum could be replaced with a more ecologically valid measure.

Moreover, additional cardiovascular measures would be suited to assess possible mediator effects that have been found to influence emotion perception within VR settings, such as arousal (Diemer et al. 2015). Here, we used EDA as an implicit autonomic measure, however, Meehan et al. (2002) suggest that greater autonomic responses due to greater presence is reflected predominantly in increased heart rate and, to a lesser degree, in skin conductance.

Another important factor that should be considered is the restricted nature of our sample. Especially when making group comparisons, in which the sample is split in half (i.e. male, female), the results do not necessarily allow for generalizations that apply to other cohorts. Especially with regard to older populations. A meta-analytic review has concluded that, overall, older adults are less accurate than young adults at recognizing emotions (Ruffman et al. 2008). However, one has to consider that many tasks used static, stereotyped photographs of emotional expression, limiting ecological validity of the task (Barrett et al. 2011). In the present study, we chose to implement a setting that induces a sense of presence in the observer, making it more similar to real-life encounters with others. However, the highly homogenous sample warrant caution when generalizing the results to wider context.

With respect to gender differences, pronounced intragroup differences in explicit as well as implicit response behavior suggest that there are individual factors that may play a more critical role in the cognitive mediation of response behavior than mere gender. Thus, future studies should aim at considering the role of individual factors, such as personality, in emotion perception studies.

## Supplementary Information

Below is the link to the electronic supplementary material.

Supplementary file1 (DOCX 24 KB)

Supplementary file2 (MP4 993 KB)

Supplementary file3 (MP4 1983 KB)

Supplementary file4 (MP4 1133 KB)

Supplementary file5 (MP4 2744 KB)

Supplementary file6 (MP4 1165 KB)

Supplementary file7 (MP4 2436 KB)

Supplementary file8 (MP4 1062 KB)

Supplementary file9 (MP4 2155 KB)
