# Supplementary material for: Does co-presence affect the way we perceive and respond to emotional interactions?
Source: Exp Brain Res. 2021 Jan 11;239(3):923–36. doi: 10.1007/s00221-020-06020-5 (PMC7943523; doi:10.1007/s00221-020-06020-5)
Supplement: Supplementary file 1 — Supplementary file1 (DOCX 24 KB) [file 221_2020_6020_MOESM1_ESM.docx]

**Supplementary Material**

| **Table S1.**  Mean values (and standard deviation) of questionnaire scores per group as well as Mann-Whitney U test displaying no significant difference between the males and females. | | | | | |
| --- | --- | --- | --- | --- | --- |
|  | Group | | Z | p | r |
|  | Female | Male |  |  |  |
| BDI-II | 5.68 ± 5.2 | 4.38 ± 3.6 | -.88 | .38 | -.10 |
| STAI_X1 | 34.56 ± 7.6 | 35.62 ± 8.0 | -.57 | .57 | -.07 |
| STAI_ X2 | 37.10 ± 9.2 | 35.73 ± 8.4 | -.46 | .65 | -.05 |
|  | | | | | |

**Table S2.** Correlation matrix between questionnaire scores and valence ratings, indicating no significant correlation.

|  | | PS_Anger | PS_Sadness | PS_Affection | PS_Happiness | VS_Anger | VS_Sadness | VS_Affection | VS_Happiness |
| --- | --- | --- | --- | --- | --- | --- | --- | --- | --- |
| Male | BDI-II | -,003 | ,059 | ,079 | ,121 | -,062 | -,021 | ,029 | -,147 |
|  | STAI_X1 | ,177 | ,251 | -,065 | ,088 | ,067 | ,153 | -,124 | -,017 |
|  | STAI_X2 | ,137 | ,241 | -,096 | -,103 | ,081 | ,143 | -,170 | -,160 |
|  |  |  |  |  |  |  |  |  |  |
| Female | BDI-II | -,266 | -,356 | ,318 | ,237 | -,083 | -,359 | ,271 | ,265 |
|  | STAI_X1 | -,056 | -,089 | ,351 | ,255 | -,104 | -,256 | ,301 | ,238 |
|  | STAI_X2 | -,385 | -,493 | ,166 | ,236 | -,276 | -,523 | ,170 | ,272 |

PS = Pictorial Scene, VS = Visual Scene

**Table S3.** Correlation matrix between questionnaire scores and explicit response behavior, indicating no significant correlation.

|  | | PS_Anger | PS_Sadness | PS_Affection | PS_Happiness | VS_Anger | VS_Sadness | VS_Affection | VS_Happiness |
| --- | --- | --- | --- | --- | --- | --- | --- | --- | --- |
| Male | BDI-II | ,184 | ,083 | -,275 | -,316 | ,128 | ,210 | -,151 | -,240 |
|  | STAI_X1 | ,017 | ,189 | ,052 | -,123 | -,075 | ,181 | ,102 | -,050 |
|  | STAI_X2 | -,032 | ,181 | ,112 | -,055 | -,150 | ,174 | ,194 | ,018 |
|  |  |  |  |  |  |  |  |  |  |
| Female | BDI-II | ,150 | ,222 | -,273 | -,210 | ,109 | ,167 | -,213 | -,262 |
|  | STAI_X1 | -,181 | ,048 | ,224 | ,094 | -,189 | -,173 | ,158 | ,036 |
|  | STAI_X2 | -,122 | ,138 | -,116 | -,015 | -,141 | -,011 | -,110 | -,095 |

PS = Pictorial Scene, VS = Visual Scene

**Table S4**. Correlation matrix between questionnaire scores and implicit response behavior, indicating no significant correlation.

|  | | PS_Anger | PS_Sadness | PS_Affection | PS_Happiness | VS_Anger | VS_Sadness | VS_Affection | VS_Happiness |
| --- | --- | --- | --- | --- | --- | --- | --- | --- | --- |
| Male | BDI-II | -,136 | -,113 | -,130 | -,267 | -,146 | ,058 | -,007 | ,068 |
|  | STAI_X1 | -,138 | ,150 | -,001 | -,171 | -,014 | ,006 | -,060 | ,020 |
|  | STAI_X2 | -,151 | -,013 | -,012 | -,237 | -,072 | ,051 | -,113 | ,153 |
|  |  |  |  |  |  |  |  |  |  |
| Female | BDI-II | -,081 | -,166 | -,198 | -,007 | ,077 | ,175 | -,172 | -,274 |
|  | STAI_X1 | ,122 | -,089 | -,055 | ,128 | ,274 | ,262 | ,022 | ,071 |
|  | STAI_X2 | ,065 | -,134 | -,151 | ,020 | ,212 | ,321 | -,146 | -,137 |

PS = Pictorial Scene, VS = Visual Scene

**Video S1**. Exemplary movie of an “affection” interaction in the pictorial condition.

**Video S2**. Exemplary movie of an “affection” interaction in the visual condition.

**Video S3**. Exemplary movie of an “anger” interaction in the pictorial condition.

**Video S4**. Exemplary movie of an “anger” interaction in the visual condition.

**Video S5**. Exemplary movie of a “happiness” interaction in the pictorial condition.

**Video S6**. Exemplary movie of a “happiness” interaction in the visual condition.

**Video S7**. Exemplary movie of a “sadness” interaction in the pictorial condition.

**Video S8**. Exemplary movie of a “sadness” interaction in the visual condition.
